# How accurate are parental responses concerning their fourth-grade children's school-meal participation, and what is the relationship between children's body mass index and school-meal participation based on parental responses?

**DOI:** 10.1186/1479-5868-9-30

**Published:** 2012-03-19

**Authors:** Amy E Paxton-Aiken, Suzanne Domel Baxter, Joshua M Tebbs, Christopher J Finney, Caroline H Guinn, Julie A Royer

**Affiliations:** 1 Institute for Families in Society, University of South Carolina, 1600 Hampton Street, Suite 507, Columbia, SC 29208, USA; 2 Department of Statistics, University of South Carolina, Columbia, SC 29208, USA

**Keywords:** Children, School, Obesity, School-meal participation, Parental response accuracy

## Abstract

**Background:**

This article investigated (1) parental response accuracy of fourth-grade children's school-meal participation and whether accuracy differed by children's body mass index (BMI), sex, and race, and (2) the relationship between BMI and school-meal participation (based on parental responses).

**Methods:**

Data were from four cross-sectional studies conducted from fall 1999 to spring 2003 with fourth-grade children from 13 schools total. Consent forms asked parents to report children's usual school-meal participation. As two studies' consent forms did not ask about lunch participation, complete data were available for breakfast on 1,496 children (51% Black; 49% boys) and for lunch on 785 children (46% Black; 48% boys). Researchers compiled nametag records (during meal observations) of meal participation on randomly selected days during children's fourth-grade school year for breakfast (average nametag days across studies: 7-35) and for lunch (average nametag days across studies: 4-10) and categorized participation as "usually" (≥ 50% of days) or "not usually" (< 50% of days). Weight and height were measured. Concerning parental response accuracy, marginal regression was used with agreement between parental responses and nametag records as the dependent variable; independent variables were BMI, age, sex, race, and study. Concerning a relationship between BMI and school-meal participation, marginal regression was used with BMI as the dependent variable; independent variables were breakfast participation, lunch participation, age, sex, race, and study.

**Results:**

Concerning breakfast participation and lunch participation, 74% and 92% of parents provided accurate responses, respectively. Parental response accuracy was better for older children for breakfast and lunch participation, and for Black than White children for lunch participation. Usual school-meal participation was significantly related to children's BMI but in opposite directions -- positively for breakfast and inversely for lunch.

**Conclusions:**

Parental response accuracy of children's school-meal participation was moderately high; however, disparate effects for children's age and race warrant caution when relying on parental responses. The BMI results, which showed a relationship between school-meal participation (based on parental responses) and childhood obesity, conflict with results from a recent article that used data from the same four studies and found no significant relationship when participation was based on nametag records compiled for meal observations.

## Background

The School Breakfast Program (SBP) and National School Lunch Program (NSLP) are federally assisted meal programs that operate in participating public and private schools. Federal regulations stipulate nutrition standards for meals that are available at full price or for free or a reduced-price for children whose families have incomes at or below the poverty level. Each school day in 2010, over 88,000 schools and 11.6 million children participated in the SBP and over 101,000 schools and 31 million children participated in the NSLP [1,2]. Assessment of the SBP showed that most schools offered breakfasts that complied with standards, although breakfasts offered tended to be lacking in fruit and whole grains; assessment of the NSLP showed that most schools offered lunches that complied with standards for protein, vitamins A and C, calcium, and iron, and exceeded standards for sodium, total fat, and saturated fat [3].

In recent years, there has been an emerging concern that participation in school-meal programs may be related to childhood obesity [3-8]. However, different studies have provided conflicting results on this topic. Specifically, studies that have relied on parental responses for school-meal participation information showed a positive association between school-lunch participation and body mass index (BMI) [9], no association between school-lunch participation and overweight status [10], and an inverse association between school-breakfast participation and BMI [11]. Results from two studies that assessed school-meal participation using daily administrative records or nametag records (compiled by research staff for direct meal observations) showed no association between school-meal participation and BMI [12,13].

An accurate assessment of school-meal participation is crucial to investigate the relationship between participation in school meals and childhood obesity. Several studies mentioned in the previous paragraph relied on parental responses of children's school-meal participation, yet past studies have shown that parents are not always acutely aware of their children's intake at school [14,15].

To our knowledge, only two studies [16,17] have investigated parental response accuracy of their children's school-meal participation; results from both studies indicated that parents might not be the most accurate source of this information. Guinn and colleagues found that, when compared to nametag records compiled by research staff for direct meal observations, 24% of parents gave incorrect responses about their fourth-grade child's usual participation in the SBP [16]. Moore and colleagues found that, when compared to administrative records, parental responses concerning school-meal participation for a short time period (day or week) overstated actual monthly and annual participation, and parental responses provided better estimates of longer-term participation in the NSLP than in the SBP [17]. Because parents are not present when their children are at school, the accuracy of parental responses concerning children's participation in school-provided meals is uncertain and may contribute to the conflicting results on the relationship between childhood obesity and school-meal participation [9-13].

For a recent article [13], Paxton and colleagues analyzed data collected from four cross-sectional dietary-reporting validation studies to investigate the relationship between fourth-grade children's BMI and school-meal participation based on nametag records compiled by research staff for direct meal observations; those results did not support a relationship. The current article used data from the same four studies to investigate parental responses of children's participation in school-provided meals to determine 1) the accuracy of parental responses concerning their fourth-grade children's participation in SBP and/or NSLP when compared to nametag records, and whether accuracy differed by children's BMI, sex, and race; and 2) after adjusting for the effects of age, sex, and race, the relationship between children's BMI and participation in SBP and NSLP during the fourth-grade school year when participation was based on parental responses.

## Methods

The University of South Carolina's institutional review board approved this secondary-analyses project. As Table 1 shows, data were collected for four cross-sectional studies during four consecutive school years with fourth-grade children from public elementary schools in one school district in Augusta, Georgia, USA. Written parental consent and child assent were obtained. Because data collection methods for each of the four studies have already been described in detail elsewhere [18-21], only a summary is provided in this article.

**Table 1:** Information on four cross-sectional studies with fourth-grade children from Augusta, Georgia, USA

	**Study A **[**19**]	**Study B **[**21**]	**Study C **[**20**]	**Study D **[**18**]
School year	1999-2000	2000-2001	2001-2002	2002-2003

Number of schools	6	11	10	6

School codes	a, b, c, d, e, f	a, b, c, d, e, g, h, i, j, k, l	a, b, c, d, e, g, h, i, j, k	b, e, f, g, k, m

Percent of children invited to participate in study who provided written child assent and parental consent^a^	73%	73%	56%	70%

Number of children in study	329	570	362	274

Questions on consent form *(response options)*	Does this child usually eat the school breakfast? *(yes, no)* Does this child usually bring breakfast from home to eat at school? *(yes, no)* Does this child usually eat the school lunch? *(yes, no)* Does this child usually bring lunch from home to eat at school? *(yes, no)*	Does this child usually eat breakfast? *(yes, no)* If yes, where? *(home, school, other)* Does this child usually eat the school lunch? *(yes, no)* Does this child usually bring lunch from home to eat at school? *(yes, no)*	Does this child usually eat breakfast? *(yes, no)* If yes, where? *(home, school, other)*	On school days, where does this child usually eat breakfast? *(at home, at school, at home and school, this child usually does not eat breakfast)*

Average number of days that nametag records^b ^were completed for breakfast (range)	35 (26-51)	26 (7-36)	16 (12-18)	7 (3-10)

Average number of days that nametag records were completed for lunch (range)^c^	10 (3-22)	7 (3-11)	4 (3-8)	5 (3-6)

Time of day when weight and height measurements were collected at school	Afternoon, after lunch	Afternoon, after lunch	Afternoon, after lunch	Morning, after breakfast

Month when weight and height measurements were collected	March	March	March	November

^a ^For each of the four studies, the percentages of Black and White girls and boys who agreed to participate were similar to those of the total population of fourth-grade children at the schools where data were collected.

^b ^Nametag records were created by research staff and were completed during school-meal observations to indicate which children (identified by the nametags they wore) participated in the School Breakfast Program and the National School Lunch Program -- that is, obtained breakfast and lunch provided by the school.

^c ^Children with fewer than 3 days of nametag records for lunch were excluded from analyses for the current article.

Schools in each study were selected from the district's 33 elementary schools to obtain a final sample of children with high participation in school-provided meals and approximately equal numbers of Black children and White children. As the schools in the studies had very few children of other races, this article's analyses excluded data for children of other races. If a child repeated fourth grade and was in more than one of the four studies, only data from the "first" fourth-grade school year were included in the analyses. During each study's school year of data collection, a mean of 59% to 70% of the children across all grades at the schools were eligible to receive free or reduced-price school meals [18-21]. The district provided school meals that complied with SBP and NSLP standards [22], and did not implement offer-versus-serve, so children could not refuse meal components. At school breakfast each day, children had a choice of a cold meal (i.e., ready-to-eat cereal, milk, graham or animal crackers, and juice or fruit) or a hot meal (e.g., sausage biscuit, milk, and juice or fruit). At school lunch each day, children had a choice of entrée.

### Participation in school-provided meals

On consent forms, questions asked parents about children's usual participation in school-provided meals. Table 1 lists questions and response options from the consent forms for each study. Although questions about usual breakfast participation were included on consent forms for all four studies, questions about usual lunch participation were included on consent forms for only the first two studies.

To document participation in school-provided meals, research staff created nametag records and completed them during direct meal observations on randomly scheduled days during children's fourth-grade school year. Table 1 shows, by study, the average number of days that nametag records were completed for breakfast and for lunch. On days when meal observations were conducted, nametags were distributed before meals to all fourth-grade children who agreed to participate in the study and were in classes scheduled for meal observation. Nametags were distributed as children entered the cafeteria (to not disrupt the cafeteria flow) and were collected immediately after meals as children left the cafeteria. Nametags were printed with large font (so readable from a distance) on cardstock that was color coded by class, laminated, and tied with yarn so children could wear nametags around their necks. Children sat at their regular places during meals. Prior to data collection, reactivity observations were conducted so that children were accustomed to wearing nametags. During meal observations, research staff indicated on nametag records which children participated in the SBP and the NSLP (i.e., obtained breakfast and lunch, respectively, that was provided by the school).

### Weight and height measurements and age

Research dietitians followed standardized procedures [23,24] to measure children's weight and height (without shoes) on digital scales (Precision Health Scale UC-300, A&D Engineering, Inc, Milpitas, CA) and portable stadiometers (Seca 214 Road Rod Portable Stadiometer, Seca Corporation, Hanover, MD), respectively. Table 1 shows the time of day and month that weight and height were measured for each study. Inter-measurer reliability was assessed daily for pairs of research dietitians on a random sample of 10% of the children; intraclass correlation reliability was > 0.99 for weight and for height for each study [25]. Each child's age at the time of measurement was calculated by subtracting his or her date of birth (obtained from school records) from the date of measurement. The Centers for Disease Control and Prevention's age/sex BMI charts were used to determine each child's BMI percentile [26]. The Expert Committee for the Prevention, Assessment, and Treatment of Child and Adolescent Overweight and Obesity recommends using BMI (kg/m^2^) to identify youth who are overweight or at risk of becoming overweight, and categorizing children as *underweight *(< 5^th ^percentile), *healthy weight *(≥ 5^th ^to < 85^th ^percentiles), *overweight *(≥ 85^th ^to < 95^th ^percentiles), *obese *(≥ 95^th ^to < 99^th ^percentiles), and *severely obese *(≥ 99^th ^percentile) [27].

## Analyses

Data analyses were performed using SAS/STAT^® ^software (Version 9.2, ^©^2002-2008, SAS Institute Inc., Cary, NC).

### Coding and analyses for parental response accuracy

Using the parental consent forms, parental responses were coded as a "1" if the parent indicated that the child usually ate the school-provided breakfast; otherwise, a "0" coding was assigned. An analogous coding assignment was used for the parental responses concerning the school-provided lunch. Similarly, using nametag records, each child's breakfast participation was coded as a "1" if the child participated in at least 50% of the days; otherwise, breakfast participation was coded as a "0." Each child's lunch participation from nametag records was categorized and coded analogously. When the parental response and the nametag record concerning a child's breakfast participation both equaled 1 or both equaled 0, this was treated as a "match." Parental responses and nametag records concerning lunch participation were treated analogously.

To model the probability of parental responses and nametag records "matching" as a function of the independent variables BMI (kg/m^2^), age (in months), sex, race (Black, White), and study (A, B, C, D), logistic regression was used. Seventy-nine children who had fewer than three days of nametag records for lunch were excluded from analyses; no children had fewer than three days of nametag records for breakfast. Marginal (first-order) logistic regression models were fit for breakfast participation and for lunch participation separately using generalized estimating equations methodology (implemented using the GENMOD procedure in SAS). This is a standard modeling approach to take when individual subjects (e.g., children) are "clustered" (e.g., within school). An independence working correlation assumption was used to calculate corrected standard errors. Large-sample Wald hypothesis tests were used to judge parameter estimates as statistically significant; when viewed individually, an estimate was declared to be significant if the associated probability value was less than 0.05.

### Analyses of the relationship between BMI and school-meal participation

Generalized estimating equation methodology was also used to fit (first-order) marginal regression models to investigate the relationship between BMI and school-meal participation based on parental responses with an independence working correlation assumption to calculate corrected standard errors. The dependent variable was BMI, and the independent variables were usual breakfast participation (yes or no based on parental responses), usual lunch participation (yes or no based on parental responses), age (in months), sex, race (Black, White), and study (A, B, C, D). Because BMI measurements were approximately symmetric in shape, a normal distribution for BMI was specified. Using different distributions did not alter the main findings. Similar to the accuracy analysis, large-sample Wald inference at a 0.05 significance level was used to judge individual effects as significant.

As two studies' consent forms did not ask about usual lunch participation, two marginal regression models were fit. Model 2A included breakfast participation and lunch participation using parental response data from the two studies that asked about both usual breakfast participation and usual lunch participation. The sample for Model 2A included 785 children. Model 2B included breakfast participation (but not lunch participation) using parental response data from all four studies. The sample for Model 2B included 1,496 children.

## Results

### Parental response accuracy

#### Summary statistics

For the 1,496 children for whom complete data on breakfast participation were available, the average BMI was 20.10 kg/m^2^, 1% were underweight, 59% were at a healthy weight, 17% were overweight, 18% were obese, and 5% were severely obese. The mean age was 10.1 years, 51% were Black, and 49% were boys. Concerning breakfast participation, there was 74% agreement (1,106/1,496) between parental responses and nametag records. Of the 390 children for whom parental responses and nametag records were in disagreement, there were 343 children for whom parents responded that their children usually did participate but the nametag records indicated that the children usually did *not *participate. For the other 47 children, parents responded that their children usually did *not *participate but the nametag records indicated that the children usually did participate.

For the subset of 785 children for whom complete data on lunch participation were available, the average BMI was 20.11 kg/m^2^, 1% were underweight, 59% were at a healthy weight, 16% were overweight, 19% were obese, and 5% were severely obese. The mean age was 10.2 years, 46% were Black, and 48% were boys. Concerning lunch participation, there was 92% agreement (721/785) between parental responses and nametag records. Of the 64 children for whom parental responses and nametag records were in disagreement, there were 4 children for whom parents responded that their children usually did participate but the nametag records indicated that the children usually did *not *participate. For the other 60 children, parents responded that their children usually did *not *participate but the nametag records indicated that the children usually did participate.

#### Regression summary

With regards to matching of parental responses and nametag records for breakfast participation, the independent variables BMI, sex, race, and study were not significant (p values > 0.07; although the result for race was borderline [p value = 0.07]). The only significant effect was due to age (p value < 0.001); specifically, the odds of matching parental responses and nametag records for breakfast participation increased significantly with children's age (holding all other variables constant). When a Bonferroni-adjusted significance criterion of 0.01 (0.05/5) was implemented for all five independent variables, the age effect remained significant.

With regards to matching of parental responses and nametag records for lunch participation, BMI (p value = 0.02), age (p value < 0.001), race (p value < 0.001), and study (p value = 0.02) were each significant. The odds of matching parental responses and nametag records for lunch participation increased with both children's BMI and age; also, parents of Black children were more accurate in responding to questions about lunch participation when compared to parents of White children, and parental responses provided for Study B were more accurate than those for Study A. Sex was not significant (p value = 0.58). When a Bonferroni-adjusted significance criterion of 0.01 (0.05/5) was implemented for all five independent variables, only age and race remained significant.

### Relationship between BMI and school-meal participation

For Model 2A (which included breakfast participation based on parental responses and lunch participation based on parental responses), a summary of the regression output is given in Table 2. Breakfast participation was related to BMI (p value = 0.05) and lunch participation was strongly related to BMI (p value = 0.001); however, the direction of the two relationships was opposite. On average, the BMI for children whose parents responded that they usually participated in breakfast was estimated to be 0.680 kg/m^2 ^*greater *than children whose parents responded that they usually did not participate in breakfast (holding all other variables constant). In contrast, on average, the BMI for children whose parents responded that they usually participated in lunch was estimated to be 1.417 kg/m^2 ^*less *than children whose parents responded that they usually did not participate in lunch (holding all other variables constant). In addition, the independent variables age and race were also strongly related to BMI (p values = 0.002 and 0.001, respectively). The average BMI was estimated to be 0.066 kg/m^2 ^greater per one-month increase in age. The average BMI for Black children was estimated to be 0.957 kg/m^2 ^greater when compared to White children. Sex was not significantly related to BMI in the presence of other independent variables (p value = 0.09). Study was not significantly related to BMI in the presence of other independent variables (p value = 0.25). When a Bonferroni-adjusted significance criterion of 0.008 (0.05/6) was implemented for all six independent variables, lunch participation, age, and race remained significant.

**Table 2:** Summary of regression output for the relationship between fourth-grade children's body mass index (BMI) and participation in school-provided meals for Model 2A^a ^(n = 785 children) and Model 2B^a ^(n = 1,496 children)

	Breakfast participation (based on parental responses)	Lunch participation (based on parental responses)	Age	Sex	Race	Study
	
	Estimate (p value)					
**BMI-Model 2A**	0.674 kg/m^2^ (0.05)	-1.417 kg/m^2^ (0.001)	0.066 kg/m^2^ (0.002)	0.539 kg/m^2^ (0.09)	0.957 kg/m^2^ (0.001)	χ^2 ^= 1.30 (0.25)

**BMI-Model 2B**	0.410 kg/m^2^ (0.06)	N/A	0.081 kg/m^2^ (< 0.001)	0.199 kg/m^2^ (0.46)	0.945 kg/m^2^ (< 0.001)	χ^2 ^= 8.96 (0.03)

^a ^Model 2A included breakfast participation and lunch participation using parental response data from the two studies that asked about both usual breakfast participation and usual lunch participation. Model 2B included breakfast participation (but not lunch participation) using parental response data from all four studies.

For Model 2B (which included breakfast participation based on parental responses but not lunch participation), a summary of the regression output is also given in Table 2. Breakfast participation was marginally related to BMI (p value = 0.06). Similar to Model 2A (which included both breakfast participation based on parental responses and lunch participation based on parental responses), age and race were strongly related to BMI (p values < 0.001). However, unlike Model 2A, for Model 2B, study was related to BMI (p value = 0.03). Sex was not significantly related to BMI in the presence of other independent variables (p value = 0.46). When a Bonferroni-adjusted significance criterion of 0.01 (0.05/5) was implemented for all five independent variables, age and race remained significant.

## Discussion

Overall, parental response accuracy of fourth-grade children's usual school-meal participation was moderately high, and better concerning lunch participation than breakfast participation. With regards to breakfast participation, responses on consent forms from approximately three in four parents on average were in agreement with nametag records compiled by researchers during direct meal observations. Parental responses concerning breakfast participation were more accurate for older children and did not differ significantly by BMI, sex, race, or study. These results for breakfast participation are similar to those in a 2002 article [16] that showed that responses from three in four parents were in agreement with nametag records and that parental response accuracy did not differ by race or sex [16]. (For that 2002 article, BMI was not included in analyses, and data were from a single study.) With regards to lunch participation, parental response accuracy was even higher at 92% agreement with nametag records. Parental responses concerning lunch participation were also more accurate for older children and differed by race; specifically, parental responses were more accurate for Black children than for White children. These disparate effects related to age and race warrant caution when relying on parental responses of children's participation in school-provided meals. In a 2009 publication, Moore and colleagues [17] also expressed caution in relying on parental responses of children's participation in school-provided meals.

Analyses indicated relationships in opposite directions between fourth-grade children's usual breakfast participation and usual lunch participation (when based on parental responses) and BMI. Specifically, when breakfast participation and lunch participation were included in the model (Model 2A), results showed an inverse relationship between school lunch participation and BMI but a (marginally significant) positive relationship between breakfast participation and BMI. When lunch participation was not in the model (Model 2B), breakfast participation remained marginally significant (and positive). These results differ from a recent article [13] that used data from the same four studies and found no significant relationship between school-meal participation (based on nametag records compiled by research staff for direct meal observations) and BMI. These results also differ from studies published in 1998 [10] and 2010 [12] that showed no significant relationship between school-meal participation (based on parental responses and daily administrative records, respectively) and BMI. In addition, these results conflict with a study published in 2009 that showed an inverse relationship between breakfast participation (based on student or parental responses) and BMI among White students [11] and with a study published in 1994 that showed a positive relationship between children who participated in school lunch (based on parental responses) and BMI [9]. These conflicting results emphasize the need to consider the source of information concerning children's school-meal participation when investigating the relationship between school-meal participation and childhood obesity.

It is possible that the conflicting results may be explained by children's food preferences, as children in the studies for this article's analyses had a choice each day of breakfast options and a choice each day of lunch entrées. Food preference data were collected for the subset of children who were interviewed for three of the four dietary-reporting validation studies which provided data for this article's analyses. However, preference data were collected only for foods observed or reported eaten during the time frame covered in the dietary recalls; thus, food preference data were not collected for all foods available on school-provided meals, no food preference data were collected for children not interviewed, and no food preference data were collected for one study (D).

It may be possible to improve parental response accuracy by rewording questions that ask parents about children's participation in school meals. For past studies, questions *(and response options) *have been worded as "Does your child eat school lunch" *(yes, no) *[9], "During the school year, about how many times a week (does the child) usually get a complete school lunch (/breakfast at school)" *(0-5) *[28], "Does (CHILD) usually eat breakfast at school under the School Breakfast Program" *(yes, no) *[29], "Does (CHILD) usually eat a complete hot lunch offered at school? Usually is defined as about 3 or more days a week" *(yes, no) *[29], and "How many days a week does (CHILD) usually eat a school lunch/breakfast" *(0-5) *[30]. Some publications have not specified the wording of the question(s) [10], or have indicated that parents were asked whether their children obtained a school breakfast and/or lunch on the most recent school day, and on each day during the most recently completed week [17]. For the four studies that provided data for the current article's analyses, questions did not define "usually", so it is possible that parents interpreted the questions differently. The authors are not aware of any research that has investigated the relationship between the wording of the questions and accuracy of parental responses. Research on this topic is needed.

The current analyses have limitations. First, the four studies from which the data arose were not designed statistically to address these research questions. Second, consent forms for only two of the four studies asked parents about children's usual participation in school lunch. Third, weight and height measurements were available for only one point in time per child rather than at the start and at the end of the fourth-grade school year. Fourth, the time of year at which parental responses regarding their child's school-meal participation was collected on consent forms (September and November of each study's school year) did not correspond with the time at which nametag records of school-meal participation were compiled (which occurred throughout each study's school year). In addition, because of the wording of questions on consent forms, it was possible to dichotomize but not to quantify parental responses of children's school-meal participation. Fifth, although childhood obesity may be related to physical activity, socioeconomic status (SES), and parental BMI, data concerning physical activity, SES, and parental BMI were not collected for the four studies. Finally, analyses for this article did not differentiate between free, reduced-price, and full-price school meals (i.e., definitions for breakfast participation and lunch participation did not change as a result of meal price); however, this was appropriate because children's participation in meals provided at school, rather than meal price, was the independent variable [7,8]. Although it is possible that the accuracy of parental responses concerning their children's participation in school-provided meals differs according to whether meals were free, reduced-price, or full price, information concerning SES at the individual child level was not collected for the four studies. However, based on the percentages of children at the schools in the four studies who were eligible to receive free or reduced-price school meals, most children in the sample were likely from families of low to moderately-low SES.

The current analyses also have several strengths. The sample for each of the four studies had approximately equal numbers of children by race (Black, White) and by sex. Results regarding the relationship between BMI and age, and between BMI and race, were similar to previous studies and national studies [31-34]. The objective measure of participation in school meals was collected during direct meal observations on nametag records compiled by research staff. Rigorous and consistent quality control procedures were implemented in all four studies for weight and height measurements. The statistical analyses specifically acknowledged the nested structure of children within school.

## Conclusions

Results from the secondary analyses in this article showed that although accuracy of parental responses concerning their fourth-grade children's school-meal participation was moderately high, accuracy differed by the meal (breakfast, lunch), age of the child, and race of the child. Results also showed conflicting relationships for breakfast and lunch between school-meal participation (based on parental responses) and children's BMI during their fourth-grade school year. These collective results warrant caution when relying on parental responses of children's participation in school-provided meals. More research, including longitudinal evidence (e.g., from first grade through fifth grade), is needed to further explore the relationship between school-meal participation and children's BMI. Research to investigate the quality of questions asked of parents concerning their children's school-meal participation may help to improve the accuracy of parental responses. School meals remain an important source of nutrition for children, and understanding their impact on childhood obesity, both positive and negative, is critical.

## Competing interests

Mrs. Paxton-Aiken has no competing interests to disclose.

Dr. Baxter's current and previous research has been funded externally by competitive grants from the National Institutes of Health as well as the United States Department of Agriculture. Dr. Baxter has served as a grant reviewer for the National Institutes of Health and the Centers for Disease Control and Prevention. Dr. Baxter is on the Board of Editors for the Journal of the American Dietetic Association (Journal of the Academy of Nutrition and Dietetics as of January, 2012).

Dr. Tebbs' current and previous research has been funded externally by competitive grants from the National Institutes of Health. Dr. Tebbs has served as a grant reviewer for the National Science Foundation.

Mr. Finney has no competing interests to disclose.

Mrs. Guinn has no competing interests to disclose.

Mrs. Royer has no competing interests to disclose.

## Authors' contributions

AEP conducted a literature review and drafted the manuscript. SDB conceived the studies, was responsible for each study's design, and helped to draft the manuscript. JMT provided oversight for statistical analyses and helped to draft the manuscript. CJF conducted all statistical analyses. CHG participated in data collection and data entry. JAR was responsible for data programming. All authors read and approved the final manuscript.

## Authors' information

AEP and CHG are Research Dietitians in the Institute for Families in Society at the University of South Carolina in Columbia, South Carolina, USA; they are Registered and Licensed Dietitians. SDB is a Research Professor in the Institute for Families in Society at the University of South Carolina in Columbia, South Carolina, USA; she is a Registered and Licensed Dietitian, and a Fellow of the American Dietetic Association (now the Academy of Nutrition and Dietetics). JMT is an Associate Professor in the Department of Statistics at the University of South Carolina in Columbia, South Carolina, USA. CJF and JAR are Research Associates in the Institute for Families in Society at the University of South Carolina in Columbia, South Carolina, USA.
